# Malignant solitary fibrous tumor of the kidney with IGF2 secretion and without hypoglycemia

**DOI:** 10.1186/s12957-024-03342-4

**Published:** 2024-07-09

**Authors:** Luting Zhou, Yang Liu, Teng Xu, Lei Dong, Xiaoqun Yang, Chaofu Wang

**Affiliations:** Department of Pathology, Shanghai Jiaotong University Medical School Affiliated Ruijin Hospital, Number 197, Ruijin Er Road, Huangpu District, Shanghai, China

**Keywords:** Solitary fibrous tumor, STAT6, *NAB2-STAT6*, Hypoglycemia, IGF2

## Abstract

**Background:**

Solitary fibrous tumor (SFT) is a rare fibroblastic mesenchymal tumor that mostly involves the pleura and infrequently involves extra-pleural sites. De novo SFT of the kidney is uncommon, and malignant SFT is extremely rare.

**Case presentation:**

We report a case of a 51-year-old man with a large malignant SFT in the left kidney. Pathological examination confirmed the diagnosis of SFT based on typical morphology, nuclear STAT6 expression, and *NAB2*-*STAT6* gene fusion. The malignant subtype was determined by a large tumor size (≥ 15 cm) and high mitotic counts (8/10 high-power fields). *KRAS* mutation was identified by DNA sequencing. Insulin-like growth factor 2 (IGF2) was diffusely and strongly expressed in tumor cells, however, hypoglycemia was not observed. Hyperglycemia and high adrenocorticotropic hormone (ACTH) concentration were observed one month after surgery. Hormone measurements revealed normal blood cortisol and aldosterone levels, and increased urinary free cortisol level. A pituitary microadenoma was identified using brain magnetic resonance imaging, which may be responsible for the promotion of hyperglycemia.

**Conclusions:**

We report a case of renal malignant SFT with a *KRAS* mutation, which was previously unreported in SFT and may be associated with its malignant behavior. Additionally, we emphasize that malignant SFT commonly causes severe hypoglycemia due to the production of IGF2. However, this effect may be masked by the presence of other lesions that promote hyperglycemia. Therefore, when encountering a malignant SFT with diffuse and strong IGF2 expression and without hypoglycemia, other lesions promoting hyperglycemia need to be ruled out.

## Introduction

Solitary fibrous tumor (SFT) is an uncommon fibroblastic tumor consisting of unorganized spindle-to-ovoid tumor cells admixed with numerous branching vessels and collagenous stroma. Nuclear STAT6 expression and specific *NAB2-STAT6* fusion aid in tumor confirmation. SFT can occur anywhere, mostly inside and partly outside the pleura. The 2020 WHO Classification of Soft Tissue and Bone Tumors classifies SFTs into benign, not otherwise specified, and malignant subtypes based on a three-variable risk model including mitotic count, patient age, and tumor size, and a modified four-variable risk model, including necrosis as a fourth variable. SFT of the kidney is uncommon [1–5], and malignant SFT is extremely rare [6–9]. Recurrent hypoglycemia has often been described in SFTs in previous reports and is caused by high levels of insulin-like growth factor 2 (IGF2) produced by the tumor [10–13].

We report a case of a large malignant SFT in the kidney that expanded its molecular profile. Based on the laboratory examination results, the preoperative diagnosis was a neuroendocrine tumor. Pathological examination confirmed the diagnosis of malignant SFT based on morphological, immunohistochemical, and molecular findings. *NAB2-STAT6* gene fusion was confirmed by RNA-based next-generation sequencing (NGS), and *KRAS* gene mutation was identified by DNA-based NGS, which has not been previously reported in SFT and may be associated with its malignant morphology. In this case, diffuse and strong IGF2 expression was observed in the tumor cells by immunohistochemistry. However, hypoglycemia was not observed in this patient. A pituitary microadenoma was identified using brain magnetic resonance imaging (MRI), which may be responsible for the promotion of hyperglycemia.

## Case presentation

**Fig. 1:**
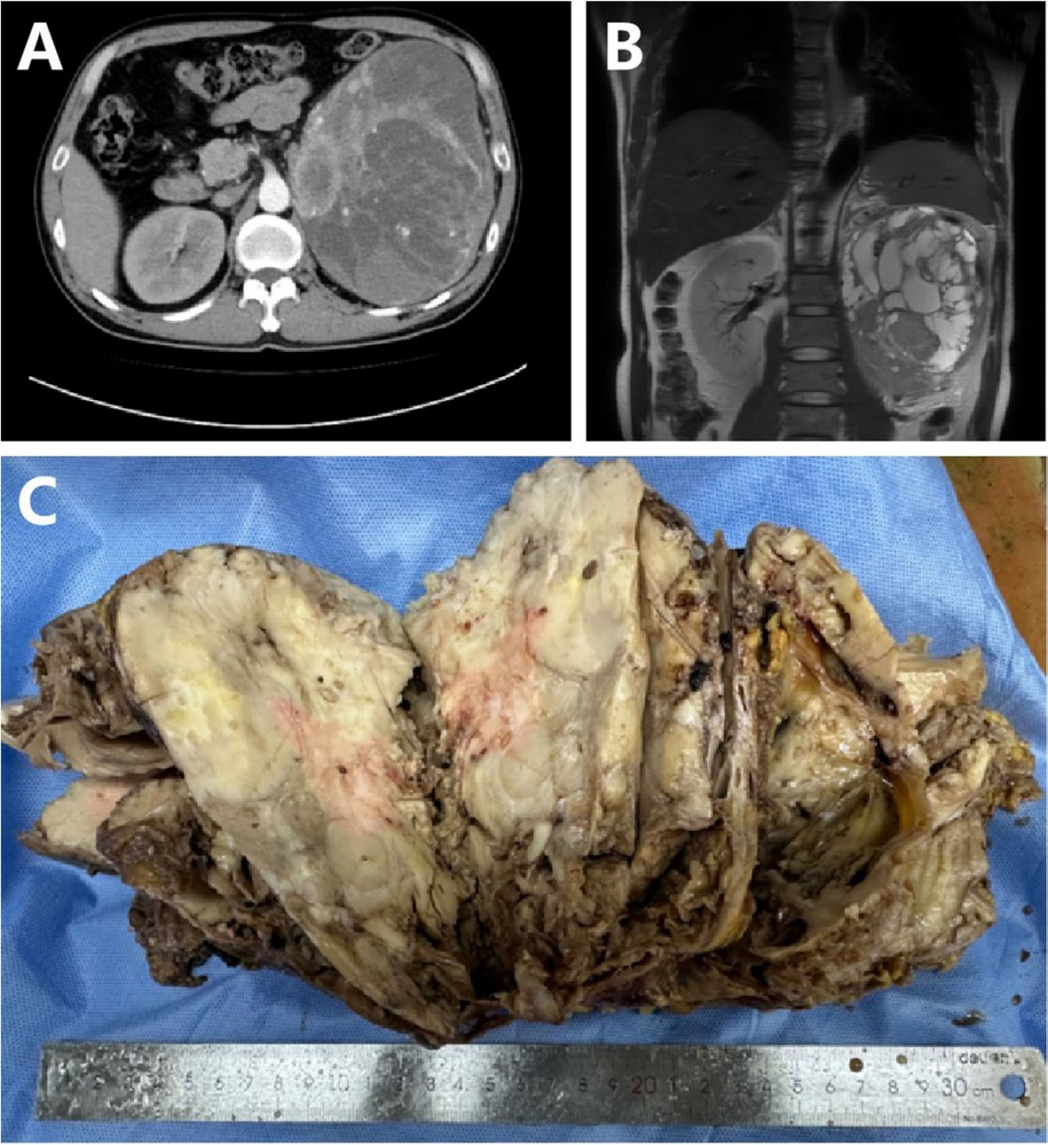
Computed tomography and magnetic resonance imaging showed a slightly enhanced, large, oval mass located at the upper pole of the left kidney (**A** and **B**). The tumor has a homogenously white and firm-cut surface (**C**)

A 51-year-old man was admitted to our hospital with foamy urine for over 12 months. Other urinary tract symptoms such as frequency, urgency, or gross hematuria were absent. The patient had experienced significant weight loss (4.0 kg) and night sweats in the last 2 months. The patient had a history of gout (duration unknown) and a 2-year history of hypertension. Routine urine tests at another hospital revealed urinary protein (3+) and occult blood. Physical examination revealed a palpable mass on the left abdomen. Laboratory examinations revealed no abnormalities except for hypokalemia (2.58 mmol/L) and hypophosphatemia (0.77 mmol/L). Computed tomography (CT) and MRI revealed a large cystic-solid mass (19×19×10 cm) occupying the left kidney without evidence of local invasion or enlarged lymph nodes (Fig. 1A and B). Hormone measurements were performed considering the history of gout, hypokalemia, and hypophosphatemia. Blood cortisol (0.90 μg/dL) was decreased and adrenocorticotropic hormone (ACTH) (67.5 pg/mL) was elevated slightly, with aldosterone, renin, angiotensin II, and androgen levels at the normal level. Urinary free cortisol levels increased, with the level of 524.62 μg/24h. An initial diagnosis of neuroendocrine tumor was made. Subsequently, the patient underwent radical nephrectomy. The surgically resected neoplasm, left kidney, ureter, and adrenal gland were sent for pathological examination.

**Fig. 2:**
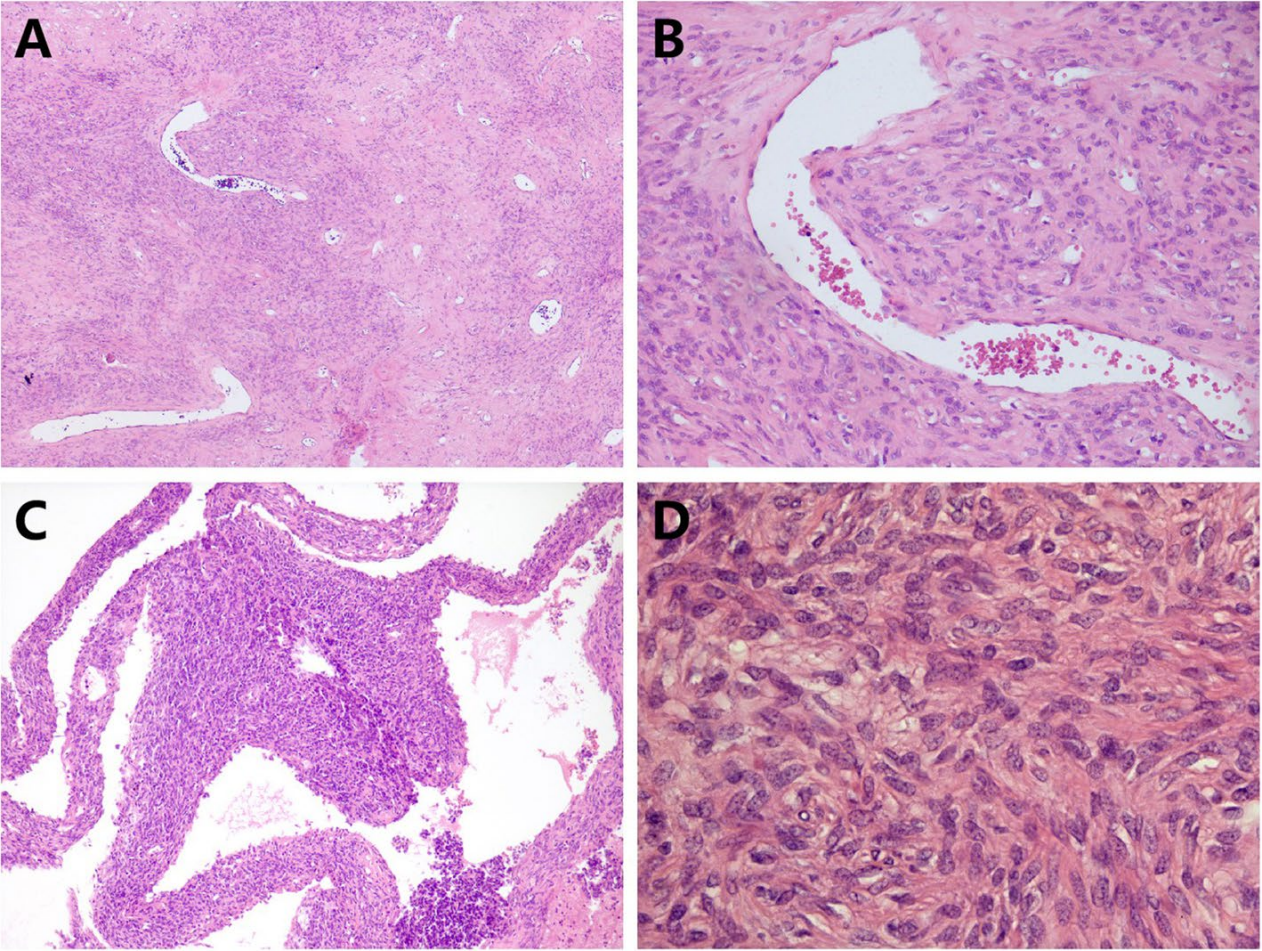
The morphology of the tumor. Microscopically, spindle tumor cells arranged haphazardly admixed with abundant collagenous stroma and numerous thin-walled branching vessels (×50) (**A**). Tumor cells had pale or eosinophilic cytoplasm and inconspicuous nucleoli (×200) (**B**). A focal cystic architecture was observed in the tumor area (×100) (**C**). A typical solitary fibrous tumor area revealed no mitotic counts in the high-power field (×400) (**D**)

**Fig. 3:**
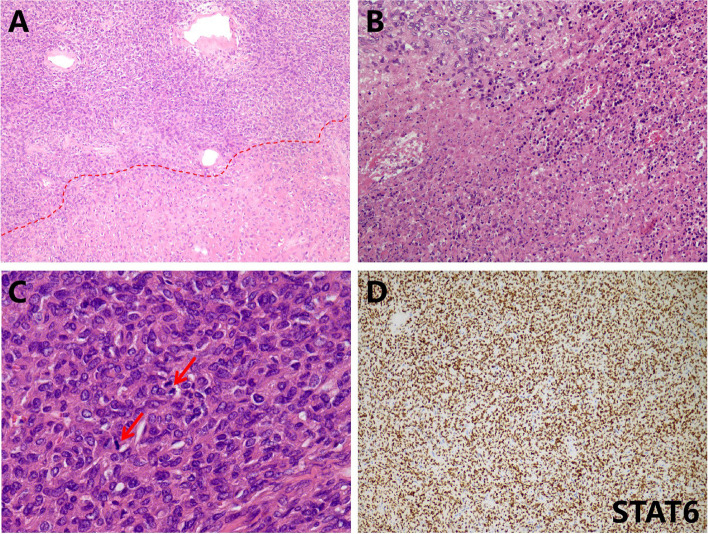
The morphology of the tumor. An abrupt transition from the lower classic solitary fibrous tumor morphology to the upper dedifferentiated morphology can be observed in focal areas (×50) (**A**). Tumor necrosis was observed (×200) (**B**). The dedifferentiated component consisted of epithelioid and spindle cells with large nuclei and pleomorphism. Mitoses were observed and indicated by arrowheads (×400) (**C**). Immunohistochemically, the tumor cells were diffusely positive for STAT6 (nuclear expression) (×100) (**D**)

**Fig. 4:**
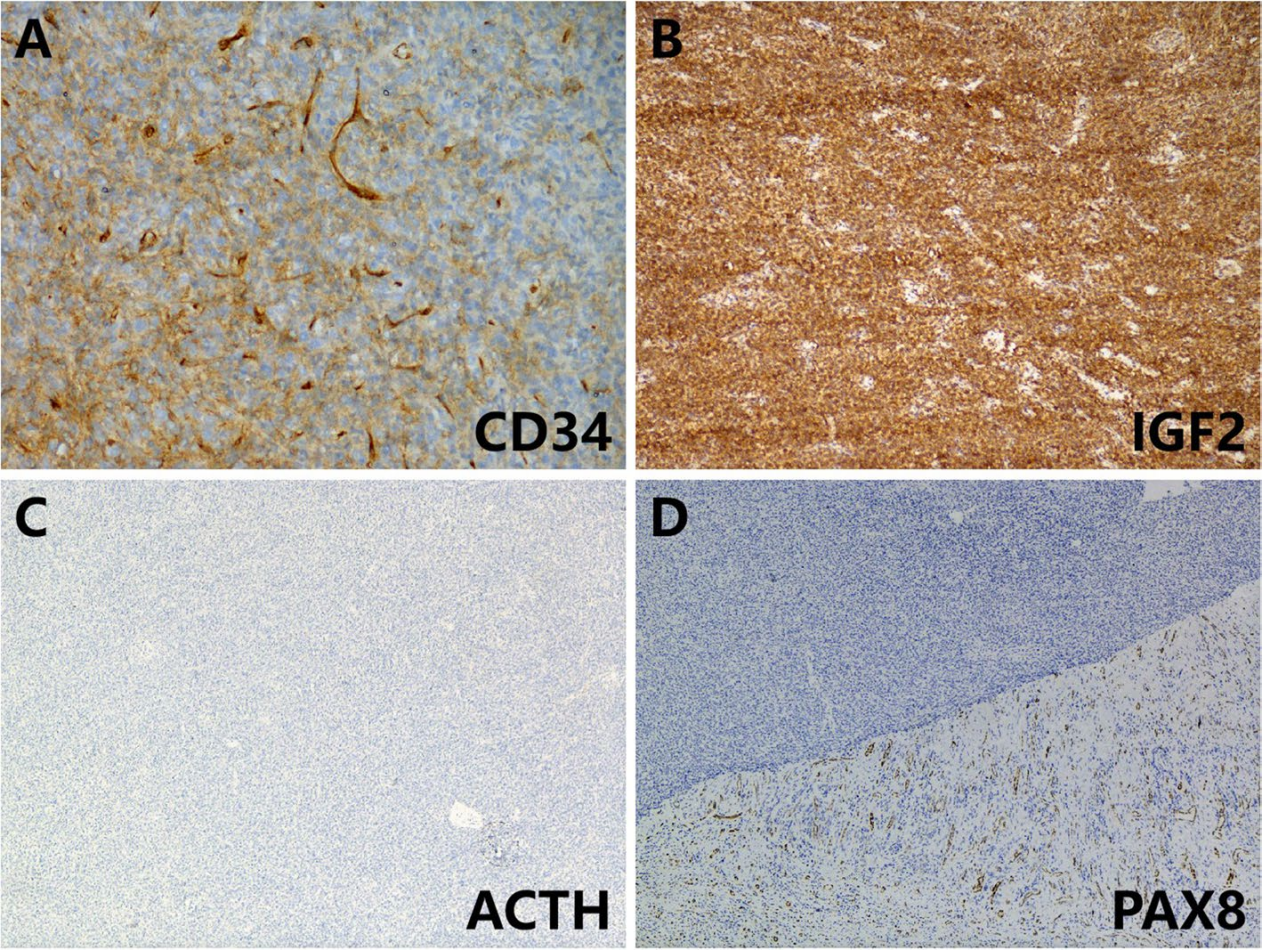
Immunohistochemically, the tumor cells were focally positive for CD34 (×200) (**A**). IGF2 was diffusely expressed in tumor cells (×50) (**B**). Tumor cells were negative for ACTH (×50) (**C**) and PAX8 (×50) (**D**) expression

**Fig. 5:**
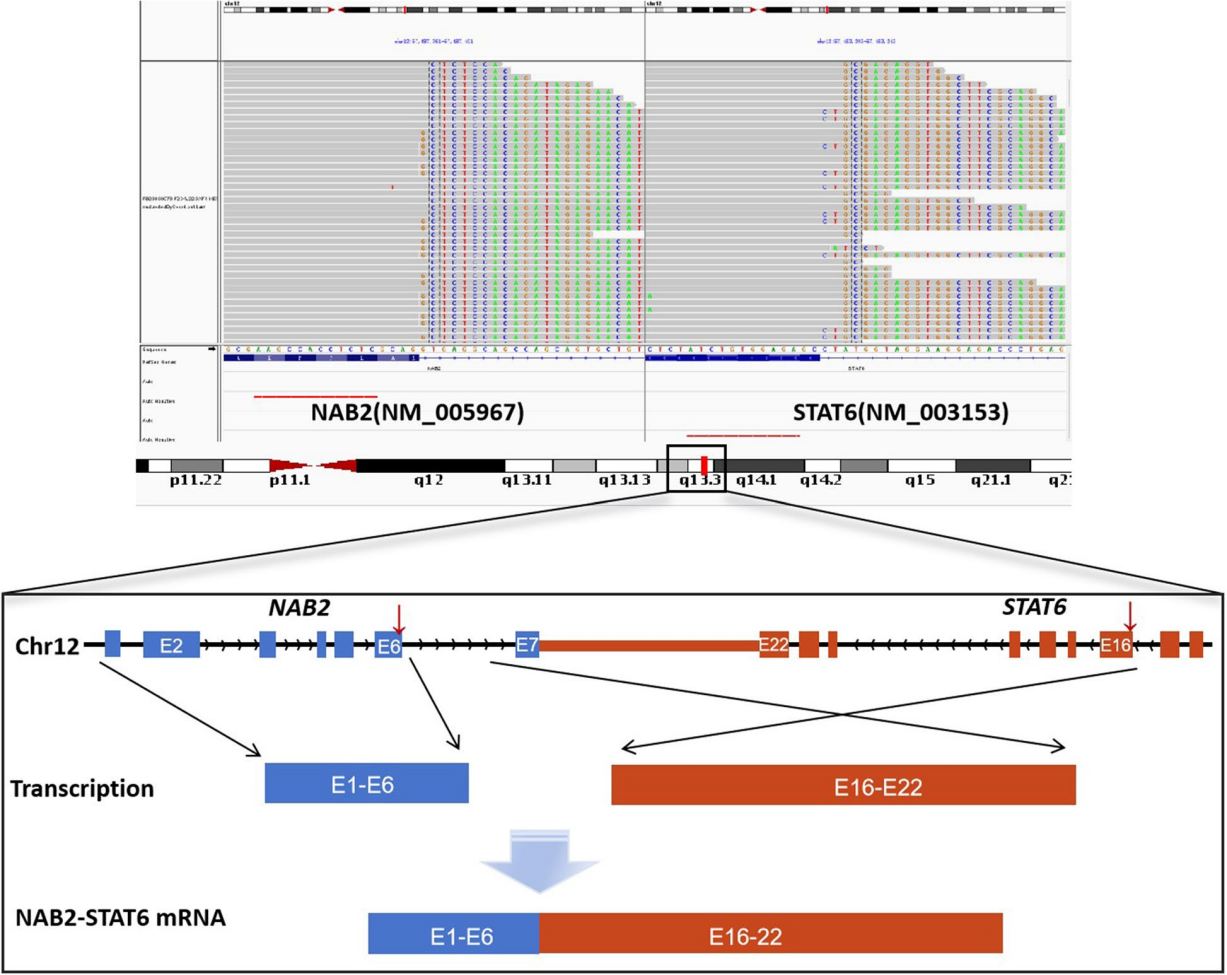
*NAB2-STAT6* fusion was identified using RNA sequencing

**Fig. 6:**
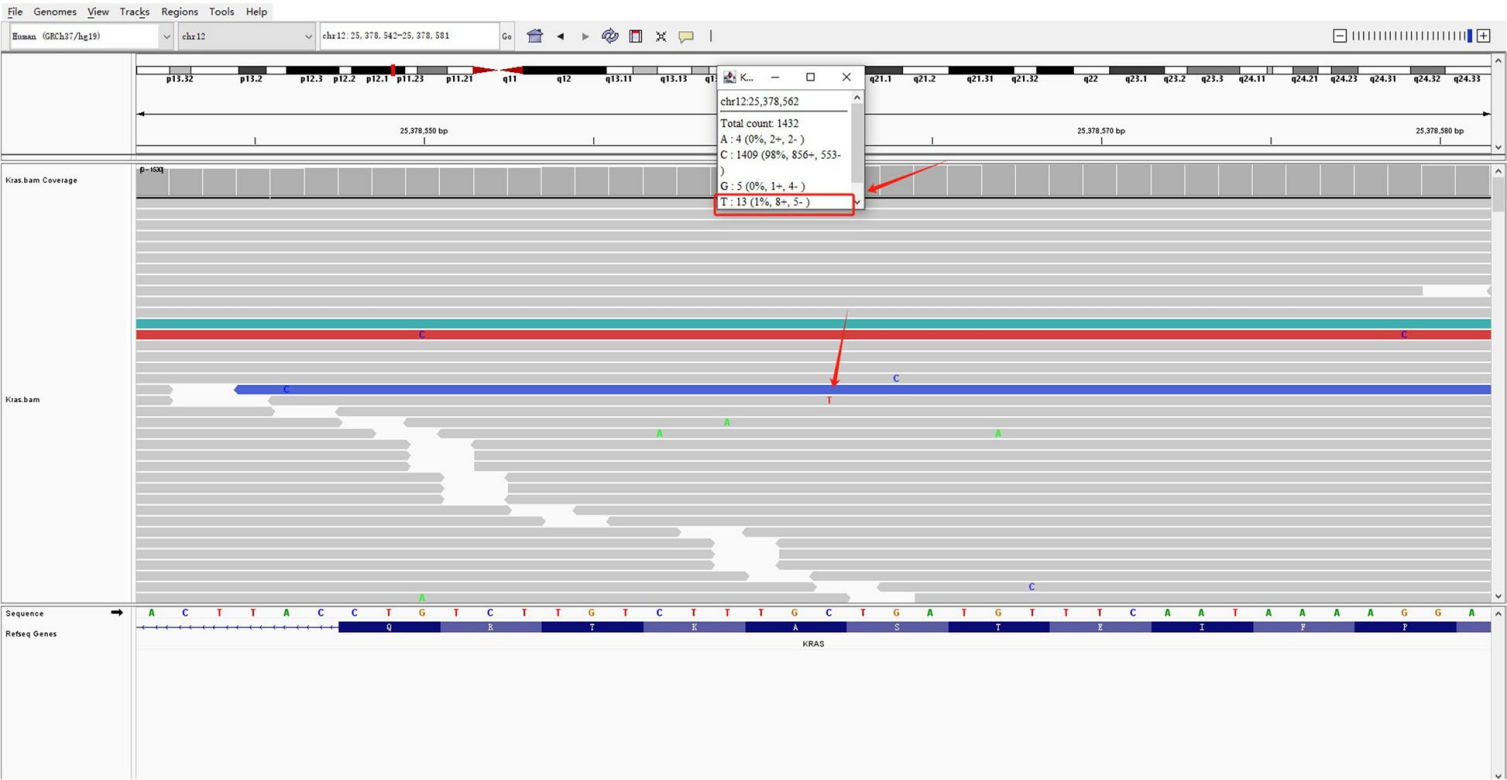
A potential *KRAS* mutation was identified using DNA sequencing

Grossly, the pathologist described an unencapsulated tumor measuring 19.0×14.0×11.0 cm, with a homogenously white and firm cut surface (Fig. 1C). Microscopically, spindle tumor cells were arranged haphazardly admixed with abundant collagenous stroma and numerous thin-walled branching vessels (Fig. 2A), indicating the diagnosis of SFT. The tumor cells had pale or eosinophilic cytoplasm and inconspicuous nucleoli (Fig. 2B). Focal cystic architecture was observed (Fig. 2C). A typical solitary fibrous tumor area revealed no mitotic counts in the high-power field (Fig. 2D). An abrupt transition from classic SFT morphology to dedifferentiated morphology was observed in the focal areas (Fig. 3A). Divergent differentiation, such as rhabdomyosarcoma or osteosarcoma, was not observed. Tumor necrosis was also observed (Fig. 3B). The dedifferentiated component consisted of epithelioid and spindle cells with large nuclei, pleomorphism, and high mitotic counts (up to 8/10 high-power fields) (Fig. 3C). Immunohistochemically, the tumor cells were diffusely positive for STAT6 (nuclear expression pattern) (Fig. 3D) and focally positive for CD34 (Fig. 4A), supporting the diagnosis of SFT. IGF2 was diffusely expressed and ACTH was negative in the tumor cells (Fig. 4B and C). PAX8 was negative in tumor cells (Fig. 4D). To further confirm the diagnosis of SFT, RNA sequencing was performed, which identified *NAB2-STAT6* fusion (Fig. 5). Considering the rarity of malignant SFT of the kidney, DNA sequencing was performed to explore its molecular features, which revealed a missense mutation in *KRAS* (exon 4 436G>A A146T) (Fig. 6). The pathological examination of the left adrenal gland revealed mild hyperplasia of the adrenal cortex.

**Fig. 7:**
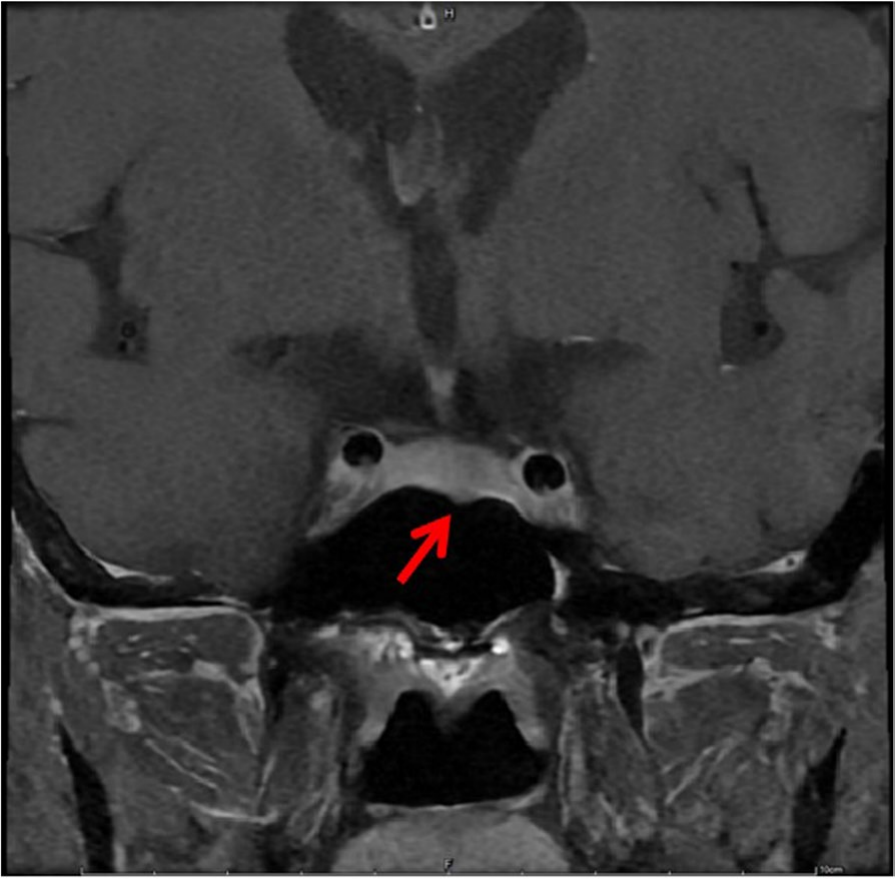
A pituitary microadenoma (0.4 cm) was identified using magnetic resonance imaging (arrowhead)

Unfortunately, we performed hormone measurements one month after surgery. Laboratory examinations validated hyperglycemia (6.86 mmol/L) and elevated ACTH concentration (150.64 mmol/L). Brain scanning was then recommended, and MRI showed a pituitary mass with the size of 0.4 cm (Fig. 7). A pituitary microadenoma secreting ACTH was suspected. However, mass resection was suspended because of the patient’s poor physical condition.

## Discussion and Conclusions

SFT is an uncommon fibroblastic tumor that occurs mostly inside and rarely outside the pleura. We report a case of a 51-year-old man with a large malignant SFT in the left kidney. The patient was diagnosed with SFT based on the typical morphology (spindle tumor cells arranged haphazardly within a collagenous stroma and branching hyalinized vasculature), nuclear STAT6 expression, and *NAB2-STAT6* fusion. The 5^th^ edition of the WHO classification introduced three- and modified four-variable risk models for the prediction of metastatic risk in SFT, including patient age, mitoses, tumor size, and tumor necrosis. Patients’ ages in years < or ≥55 are assigned 0 or 1 point. Mitoses per 10 HPFs of 0, 1-3, or ≥4 are assigned 0, 1, or 2 points, respectively. Tumor sizes of <5 cm, <10 cm, <15 cm, or ≥15 cm are assigned 0, 1, 2, or 3 points. The low-, intermediate-, and high-risk SFT ranged 0-2, 3-4, and 5-6, respectively. In the current study, the malignant subtype was confirmed based on sarcoma morphology in focal areas, large tumor size, high mitotic counts, and tumor necrosis, despite no local invasion or distant metastasis.

Immunohistochemically, CD34 expression was lost in most of the tumor areas in this patient. Although SFT typically exhibits a strong and diffuse staining pattern for CD34, as described in previous studies, its expression can be lost in dedifferentiated areas [14]. Nuclear STAT6 expression exhibited a strong diffuse pattern in tumor cells. However, STAT6 expression has also been reported in other tumors such as well-differentiated or dedifferentiated liposarcomas and desmoid tumors [15, 16]. Considering that the tumor was located in the left kidney, sarcomatoid renal cell carcinoma also needed to be excluded. The epithelial marker AE1/AE3 or renal origin marker PAX2/PAX8 was negative in the tumor cells, which can aid in the differential diagnosis. However, previous studies have reported PAX2 and PAX8 expression in SFT [17]. In this instance, SFT with PAX2/PAX8 expression may be misleading in the diagnosis of sarcomatoid renal cell carcinoma, which is a potential diagnostic pitfall.

*KRAS* mutations have been reported in cancers of several organs [18]. However, *KRAS* mutations have not been described in SFT. To our knowledge, this study is the first study to describe *KRAS* mutations in a malignant SFT of the kidney. Considering that *KRAS* mutations are usually associated with aggressive behavior and poor prognosis [19], *KRAS* mutation may be responsible for the malignant subtype of this tumor.

Previous studies have shown that malignant SFTs are often associated with stubborn hypoglycemia [10–13], which is caused by IGF2 produced by the tumor. Circulated IGF2 levels may have been normalized after tumor resection, and severe hypoglycemia may have been resolved. However, we did not observe hypoglycemic symptoms in the patient, although diffuse and strong IGF2 expression was observed in the tumor cells using IHC. In contrast, hyperglycemia was present one month after surgery. Brain scanning was recommended because of the elevated ACTH levels. A pituitary mass was finally identified, which may be responsible for the elevated blood ACTH level, hyperglycemia, and adrenal cortex hyperplasia, although mass resection was not performed because of poor physical condition.

In conclusion, we introduced a malignant SFT of the kidney with a confirmed *NAB2-STAT6* fusion and a novel *KRAS* mutation. Mutated *KRAS* may be associated with malignant behavior. We observed diffuse and strong IGF2 expression, however, hypoglycemia was not observed. We postulated the balance between SFT that produces IGF2, which can promote hypoglycemia, and pituitary adenoma that secretes ACTH, which can promote hyperglycemia, might underlie the clinical course of the patient. Therefore, caution should be exercised when encountering a malignant SFT with diffuse and strong IGF2 expression and normal blood glucose levels. In this instance, other lesions promoting glucose levels need to be excluded.

## Data Availability

The datasets used and/or analysed during the current study are available from the corresponding author on reasonable request.

## Abbreviations

SFT: Solitary fibrous tumor
IGF2: Insulin-like growth factor 2
NGS: Next-generation sequencing
CT: Computed topography
MRI: Magnetic resonance imaging
ACTH: Adrenocorticotropic hormone
